# Neutrophil ratio trajectory and the short-term outcome of perinatal hypoxic-ischemic encephalopathy

**DOI:** 10.3389/fped.2025.1555981

**Published:** 2025-06-17

**Authors:** Yunxia Li, Yujie Lang, Ke Deng, Dongmei Zhou, Lili Kang, Xiaoying Li

**Affiliations:** ^1^ Department of Neonatology, Jinan Children’s Hospital, Jinan, Shandong, China; ^2^ Department of Neonatology, Children’s Hospital Affiliated to Shandong University, Jinan, Shandong, China

**Keywords:** perinatal hypoxic-ischemic encephalopathy, neutrophil ratio, trajectory analysis, nonlinear, short-term outcome

## Abstract

**Background:**

Perinatal hypoxic-ischemic Encephalopathy (HIE) is a disease of high mortality and morbidity. The neutrophil ratio (NR), a key indicator, is widely used in clinical practice. However, the relationship between dynamically changing NR and the short-term prognosis of HIE is unclear. We aimed to explore the dynamic changes and potential classifications of NR over time using trajectory analysis and to investigate its relationship with the short-term prognosis of HIE.

**Methods:**

We performed a retrospective analysis of medical records from patients at Shandong University Affiliated Children's Hospital from 2014 to 2022. NR trajectories were modeled in 605 HIE infants using latent growth mixture modeling from 0 to 4 weeks. Then using logistic regression analyze the relationship between NR trajectories and the short-term outcome of HIE. Finally, restricted cubic spline regression model were used to assess the nonlinear relationship between baseline NR and the outcome.

**Results:**

Two distinct NR trajectories could be modeled, a descending (class 1) and a ascending group (class 2). The ascending group (class 2) was associated with the poor outcome (OR 5.72, 95% CI 2.97–10.87, *P* < 0.001; OR_adj_ 4.78, 95% CI 2.42–9.28, *P* < 0.001). A significant nonlinear relationship between NR and the risk of poor outcomes (*F* = 13.16, *P* < 0.001), potentially exhibiting a U-shaped relationship.

**Conclusion:**

The ascending NR group was strongly associated with poorer short-term outcomes. And both low and high NRs at baseline were associated with increased risk of poorer short-term outcomes.

## Introduction

Perinatal hypoxic-ischemic Encephalopathy (HIE) is defined as a type of brain dysfunction that occurs when an infant's brain does not receive enough oxygen and blood flow around the time of birth (1, 2). HIE affects approximately one to eight per 1,000 live newborns worldwide (3), and is one of the leading causes of persistent central nervous system injury, resulting in significant newborn mortality or neurodevelopmental impairment (4). Infants with moderate HIE have a 10% mortality rate, while 30% of survivors experiencing impairments (5). For severe HIE, the mortality rate climbs to 60%, with nearly all survivors suffering from epilepsy and neurodevelopmental issues such as cerebral palsy and intellectual disability (6).

Neutrophils play an important role in the body's immune response and inflammation. Some clinical analysis studies have shown that neutrophils were associated with HIE (7–14), including poorer neurological outcomes and aggravation of brain injury (10). Additionally, some animal experiments (13, 15–19) have explored the mechanisms through which neutrophils contribute to HIE. Neutrophils as the most abundant leukocytes in circulation and the first peripheral immune cells to reach the injured brain were highly involved in regulating neuroinflammatory responses and neuronal apoptosis after brain injury (15, 19, 20).

However, these clinical analysis studies typically measured neutrophils at a single time point (e.g., on admission or at the peak of illness severity), ignoring their dynamic changes over time. Research on the dynamic changes of neutrophils in relation to HIE appeared to be scarce. The neutrophil ratio (NR), which was the ratio of neutrophils to the total number of white blood cells, serves as a key indicator among neutrophil-related metrics. It was widely used in clinical practice because it accounts for individual differences more effectively than absolute value indicators. Therefore, this study aimed to explore the dynamic changes and potential classifications of NR over time using trajectory analysis and to investigate its relationship with the short-term prognosis of HIE.

## Methods

### Study population and data source

A retrospective analysis was performed of patients who diagnosed with perinatal HIE at Shandong University Affiliated Children's Hospital, over the period 2014 to 2022. Electronic medical records (EMRs) from this period served as the primary data source.

### Data collection

Patient information was extracted from EMRs on December, 2023. Data included demographic information (such as gender, gestational age, age at admission), maternal pregnancy information (including maternal complications, medications, delivery mode, placental condition, and amniotic fluid status), and general infant information (such as postnatal asphyxia, Apgar scores, etc.). NR was collected for each patient from admission to discharge. Multiple laboratory examination data points were included.

### Data preprocessing

Because more than 90% of the time of NR detection occured within the first 4 weeks of life, we selected NR data detected within the first 3 weeks of life for analysis. Age in weeks was used for trajectory analysis model as the time unit. For each age week, the mean value of multiple tests conducted was calculated, and the age in days was recorded as the minimum value.

**Figure 1:**
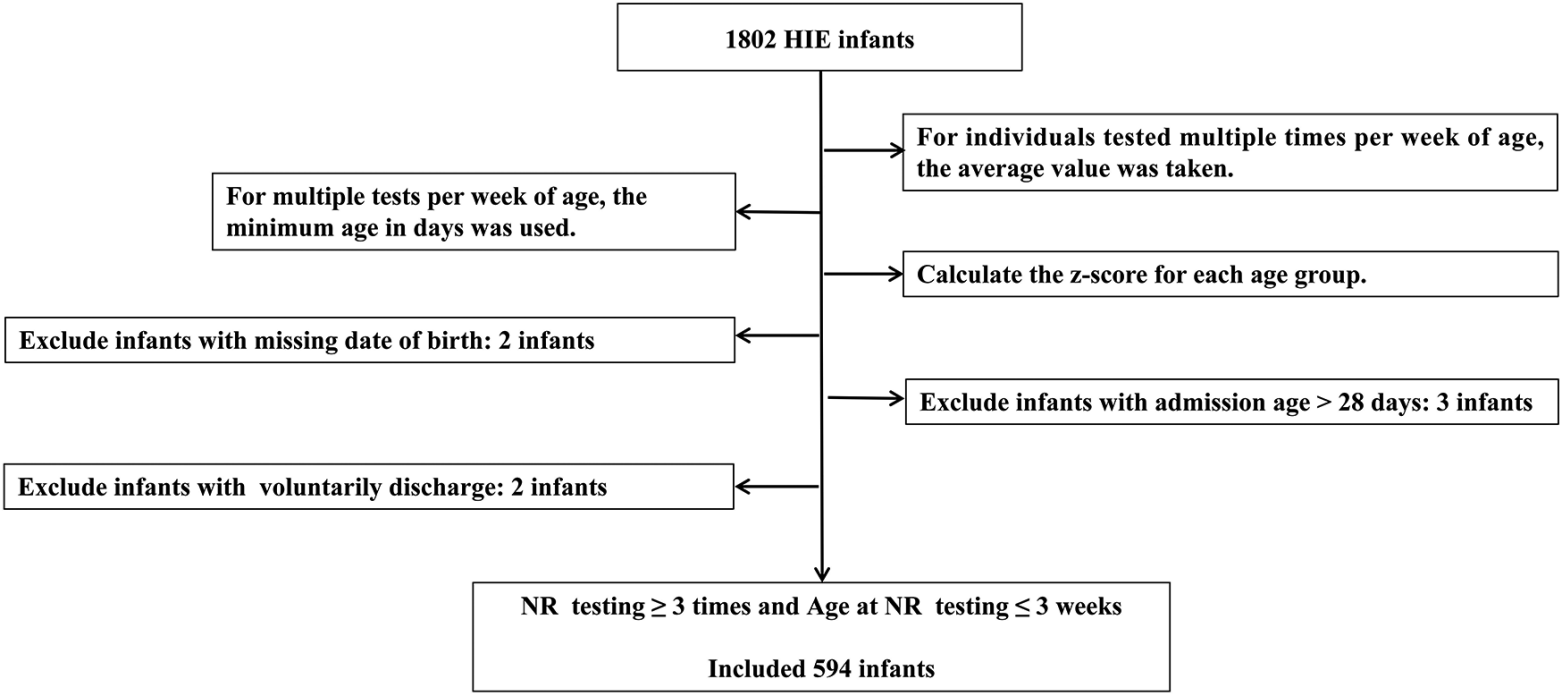
Data processing flowchart.

Age intervals were standardized based on age points at 3, 7, and 14 days. Excluded infants with missing birth dates, those admitted after 28 days of age, or those who voluntarily discharge. Voluntarily discharge was defined as the withdrawal of treatment by the guardian for various reasons and discharge against medical advice. The flow chart of data processing is shown in Figure 1.

Missing baseline demographic data, such as gestational age and birth weight, were imputed using a random forest algorithm. The performance of the imputation was evaluated using root mean squared error (RMSE) and mean absolute error (MAE).

### Outcome

The discharge status of the patient was used as the outcome variable of short-term prognosis. Cure and improvement were defined as good outcomes, while uncure and death were defined as poor outcomes.

Cure was defined as the complete resolution of symptoms, absence of clinical abnormalities, normal neurological reflexes and muscle tone, and no notable abnormalities on neuroimaging, or only minor changes that do not affect function. Patients meeting the criteria for cure required no special follow-up, aside from routine developmental monitoring.

Improvement was defined as a significant reduction in symptoms with potential mild neurological abnormalities or developmental delay, partial improvement of lesions on neuroimaging with minor residual abnormalities, and neurological examination findings that may show mild deviations without substantial impact on daily life. Patients in the improvement category typically required regular follow-up to assess neurological development.

Uncure was defined as the persistence of symptoms or only partial relief with notable neurological abnormalities, such as seizure activity, severe muscle tone abnormalities, or marked developmental delay. Neuroimaging findings in this group typically showed significant lesions with no evident improvement, indicating ongoing brain injury. Neurological examination often revealed significant abnormalities in reflexes and muscle tone, potentially impacting daily life and development. These patients generally required continued medical intervention and close follow-up. Death was defined as mortality during hospitalization due to HIE progression or related complications.

### Statistical analysis

#### Trajectory analysis

The Latent Growth Mixture Modeling (LGMM) (21) was employed to explore the temporal patterns of NR changes and identify distinct latent growth trajectory groups. LGMM can identify latent heterogeneity within the data by assigning individuals to different latent classes, each characterized by a unique growth trajectory. Individuals are assigned a probability of belonging to each class and then assigned to the class for which they have the highest probability of membership. If a subject did not have any measurements in a specific time period, they were excluded from contributing to the estimation of the mean trajectory and not allocated a class for that age range. If a subject had 1 or more measurements in a specific time period, their information did contribute to the calculation of mean trajectories in that age range using full information maximum likelihood.

Based on mean posterior probabilities >0.7 for each group, a group size >5.0%, and the lowest Bayesian Information Criterion (BIC), the optimal number of trajectory groups and the best-fit model were determined (22).

#### Logistic regression analysis

Primary analysis was conducted using the imputed data. For continuous data, normality was assessed using a Shapiro–Wilk test. Data conforming to a normal distribution were described using mean ± standard deviation, and inter-group comparisons were conducted using *t*-tests. Skewed data were described using the median, and inter-group comparisons were performed using Wilcoxon rank sum test. Count data were described using frequencies and proportions, and inter-group comparisons were evaluated using the chi-square test or Fisher's exact test.

Logistic regression models were developed: Model 1 included trajectory groups as predictors without adjusting for other variables, while Model 2 adjusted for significant variables identified in the univariate analysis.

Restricted cubic spline regression models were used to assess the nonlinear relationship between baseline NR and the outcome. The number of nodes was determined based on the minimum Bayesian Information Criterion (BIC), and dose-response and nonlinear curves were plotted accordingly.

#### Sensitivity analysis

In sensitivity analysis, we repeated the analysis using the non-imputed data to observe consistency with the imputed data results and assess the stability of the results.

Statistical analyses were performed using R version 4.3.1, and statistical significance was defined as a *P* value of 0.05.

## Results

### Developmental trajectories

**Figure 2:**
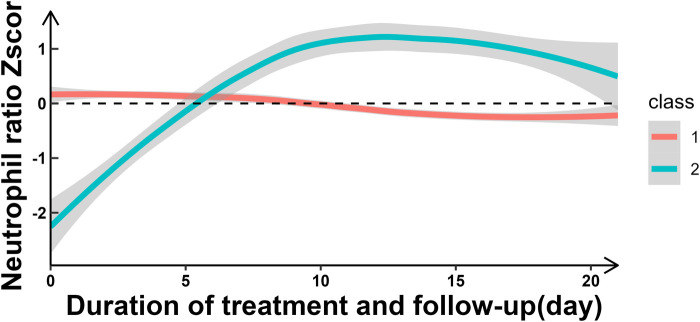
Trajectories of NR with 2 class.

Two distinct NR trajectories could be modeled, each with an average posterior probability greater than 0.7 (0.93 and 0.82, respectively). The trajectory group sizes were all greater than 5% (87.04% and 12.96%, respectively), and the BIC was minimum. These two NR trajectories were classified as the slowly declining stable group (class 1-red line) and ascending group (class 2-green line) (Figure 2, Table 1).

**Table 1:** BIC and posterior probability for different numbers of classes of trajectories.

Model	BIC	Class (%)					Average posterior probability				
		Class 1	Class 2	Class 3	Class 4	Class 5	Prob1	Prob2	Prob3	Prob4	Prob5
1	5816.61	100.00									
2	5739.05	86.49	13.51				0.94	0.88			
3	5753.03	6.92	14.17	78.91			0.65	0.86	0.88		
4	5770.66	62.27	16.97	11.20	9.56		0.77	0.68	0.79	0.64	
5	5797.24	49.92	11.20	17.63	10.05	11.20	0.65	0.63	0.71	0.79	0.58

### Baseline demographics

In the full cohort (*n* = 594, 65.82% were male, median birth weight (BW) was 3.10 kg [interquartile range (IQR) 2.60–3.50], median gestational age (GA) was 38.71 (37.14–39.86) weeks, 28.28% had pregnancy complications, 26.94% had a medication history during pregnancy, 24.58% were firstborns, 34.18% were first deliveries, 23.06% was premature, the median 5-minute Apgar scores were 7 (5–8), 68.69% were delivered by cesarean section, 60.10% had intrauterine distress, 18.69% had premature rupture of membranes, 53.03% had amniotic fluid contamination or bloody amniotic fluid, 11.62% had abnormal placentas, the median ages of the fathers and mothers were 30 (27–35) and 30 (27–34) years respectively, 69.02% lived in village, 13.97% received hypothermia therapy, and the median hospital stay was 19 (15–25) days (Table 2).

**Table 2:** Participants’ characteristics by trajectory groups.

Variable	Main analysis				Sensitivity analysis (non-imputed data)			
	Good (*n* = 548)	Poor (*n* = 46)	Total (*n* = 594)	*P*	Good (*n* = 548)	Poor (*n* = 46)	Total (*n* = 594)	*P*
Trajectory class								
Slowly declining stable group	488 (89.05)	27 (58.70)	515 (86.70)	<0.001	488 (89.05)	27 (58.70)	515 (86.70)	<0.001
Ascendin group	60 (10.95)	19 (41.30)	79 (13.30)		60 (10.95)	19 (41.30)	79 (13.30)	
Gender								
Male	360 (65.69)	31 (67.39)	391 (65.82)	0.94	360 (65.69)	31 (67.39)	391 (65.82)	0.94
Female	188 (34.31)	15 (32.61)	203 (34.18)		188 (34.31)	15 (32.61)	203 (34.18)	
Birth weight (kg)	3.10 (2.60–3.50)	2.67 (2.11–3.10)	3.10 (2.60–3.50)	<0.001	3.10 (2.62–3.50)	2.65 (2.02–3.10)	3.10 (2.60–3.50)	<0.001
Gestational age (weeks)	38.71 (37.29–39.86)	38.21 (36.14–39.54)	38.71 (37.14–39.86)	0.06	38.71 (37.29–39.89)	38.14 (36.14–39.57)	38.57 (37.14–39.86)	0.06
Sepsis								
Yes	217 (39.60)	21 (45.65)	238 (40.07)	0.52	217 (39.60)	21 (45.65)	238 (40.07)	0.52
No	331 (60.40)	25 (54.35)	356 (59.93)		331 (60.40)	25 (54.35)	356 (59.93)	
Pregnancy complications								
Yes	159 (29.01)	9 (19.57)	168 (28.28)	0.23	159 (29.01)	9 (20.45)	168 (28.38)	0.30
No	389 (70.99)	37 (80.43)	426 (71.72)		389 (70.99)	35 (79.55)	424 (71.62)	
Medicationduring pregnancy								
Yes	151 (27.55)	9 (19.57)	160 (26.94)	0.32	151 (27.71)	9 (20.45)	160 (27.16)	0.39
No	397 (72.45)	37 (80.43)	434 (73.06)		394 (72.29)	35 (79.55)	429 (72.84)	
Parity								
1	134 (24.45)	12 (26.09)	146 (24.58)	0.65	134 (24.45)	12 (27.27)	146 (24.66)	0.39
2	179 (32.66)	12 (26.09)	191 (32.15)		179 (32.66)	10 (22.73)	189 (31.93)	
≥3	235 (42.88)	22 (47.83)	257 (43.27)		235 (42.88)	22 (50.00)	257 (43.41)	
Number of deliveries								
1	189 (34.49)	14 (30.43)	203 (34.18)	0.71	189 (34.49)	14 (31.82)	203 (34.29)	0.67
2	275 (50.18)	23 (50.00)	298 (50.17)		275 (50.18)	21 (47.73)	296 (50.00)	
≥3	84 (15.33)	9 (19.57)	93 (15.66)		84 (15.33)	9 (20.45)	93 (15.71)	
Prematurity								
Yes	122 (22.26)	15 (32.61)	137 (23.06)	0.16	122 (22.26)	15 (33.33)	137 (23.10)	0.13
No	426 (77.74)	31 (67.39)	457 (76.94)		426 (77.74)	30 (66.67)	456 (76.90)	
Apgarscore 1 min	6 (3–8)	5 (3–7)	6 (3–8)	0.10	6 (3–8)	5 (3–7)	6 (3–8)	0.38
Apgarscore 5 min	7 (5–8)	7 (5–8)	7 (5–8)	0.77	7 (5–8)	7 (5–8)	7 (5–8)	0.57
Apgarscore 10 min	8 (7–9)	7 (6–8)	8 (7–9)	0.21	8 (6–9)	7 (6–8)	8 (6–9)	0.28
Delivery type								
C-section	378 (68.98)	30 (65.22)	408 (68.69)	0.72	378 (68.98)	29 (64.44)	407 (68.63)	0.64
SVD	170 (31.02)	16 (34.78)	186 (31.31)		170 (31.02)	16 (35.56)	186 (31.37)	
iUGR								
Yes	330 (60.22)	27 (58.70)	357 (60.10)	0.96	327 (60.67)	26 (60.47)	353 (60.65)	1.00
No	218 (39.78)	19 (41.30)	237 (39.90)		212 (39.33)	17 (39.53)	229 (39.35)	
Premature rupture of fetal membranes								
Yes	105 (19.16)	6 (13.04)	111 (18.69)	0.41	105 (19.34)	6 (13.64)	111 (18.91)	0.47
No	443 (80.84)	40 (86.96)	483 (81.31)		438 (80.66)	38 (86.36)	476 (81.09)	
Amniotic fluid								
Normal	258 (47.08)	21 (45.65)	279 (46.97)	0.97	243 (46.37)	18 (42.86)	261 (46.11)	0.78
Abnormal (bloodyorpolluted)	290 (52.92)	25 (54.35)	315 (53.03)		281 (53.63)	24 (57.14)	305 (53.89)	
Placenta								
Normal	484 (88.32)	41 (89.13)	525 (88.38)	1.00	471 (88.20)	39 (88.64)	510 (88.24)	1.00
Abnormal	64 (11.68)	5 (10.87)	69 (11.62)		63 (11.80)	5 (11.36)	68 (11.76)	
Father age	30 (27–35)	30 (28–34)	30 (27–35)	0.78	31 (27–35)	30 (28–34)	31 (27–35)	0.75
Mother age	30 (27–34)	31 (28–34)	30 (27–34)	0.17	30 (27–34)	31 (28–34)	30 (27–34)	0.18
Environment during pregnancy								
City	175 (31.93)	9 (19.57)	184 (30.98)	0.11	174 (31.87)	9 (20.45)	183 (31.02)	0.16
Village	373 (68.07)	37 (80.43)	410 (69.02)		372 (68.13)	35 (79.55)	407 (68.98)	
Hospitaldays	19 (15–24)	21 (14–32)	19 (15–25)	0.19	19 (15–24)	21 (14–32)	19 (15–25)	0.26
Hypothermia treatment								
Yes	79 (14.42)	4 (8.70)	83 (13.97)	0.39	79 (14.42)	4 (8.70)	83 (13.97)	0.39
No	469 (85.58)	42 (91.30)	511 (86.03)		469 (85.58)	42 (91.30)	511 (86.03)	

### Baseline demographics by outcome groups

The differences between the outcome groups (good/poor) in trajectory class, birth weight (kg) and gestational age (weeks) were statistically significant (*P* < 0.05). Other variables were not statistically significant (Table 2).

The ascending group comprises 10.95% and 41.30% of the good and poor group, respectively. The median BW was 3.10 (2.63–3.50) kg and the median GA was 38.71 (37.29–39.86) weeks for the good group, 2.67 (2.11–3.10) kg and 38.21 (36.14–39.54) weeks for the poor. Prematurity account for 22.26% and 32.61% of the good and poor group, respectively.

### Logistic regression results

Compared to class 1 (“slowly declining stable group”), class 2 (“ascending group”) was associated with the poor outcome (OR 5.72, 95% CI 2.97–10.87, *P* < 0.001) (Table 3, Supplementary Tables S1, S2), without adjustment. With adjusted for GA, BW and sepsis, class 2 (“ascending group”) was also associated with the poor outcome (OR 4.78, 95% CI 2.42–9.28, *P* < 0.001) (Table 3, Supplementary Table S3).

**Table 3:** Results of logistic regression regarding trajectory groups.

Trajectory class	Model 1		Model 2	
	OR (95% CI)	*P*	OR (95% CI)	*P*
Slowly declining stable group (Class 1)	1	-	1	-
Ascending group (Class 2)	5.72 (2.97, 10.87)	<0.001	4.78 (2.42, 9.28)	<0.001

Model 1, not adjusted for any covariates; Model 2, adjusted for GA, BW and sepsis.

### Nonlinear relationship between baseline NR and the outcomes

**Figure 3:**
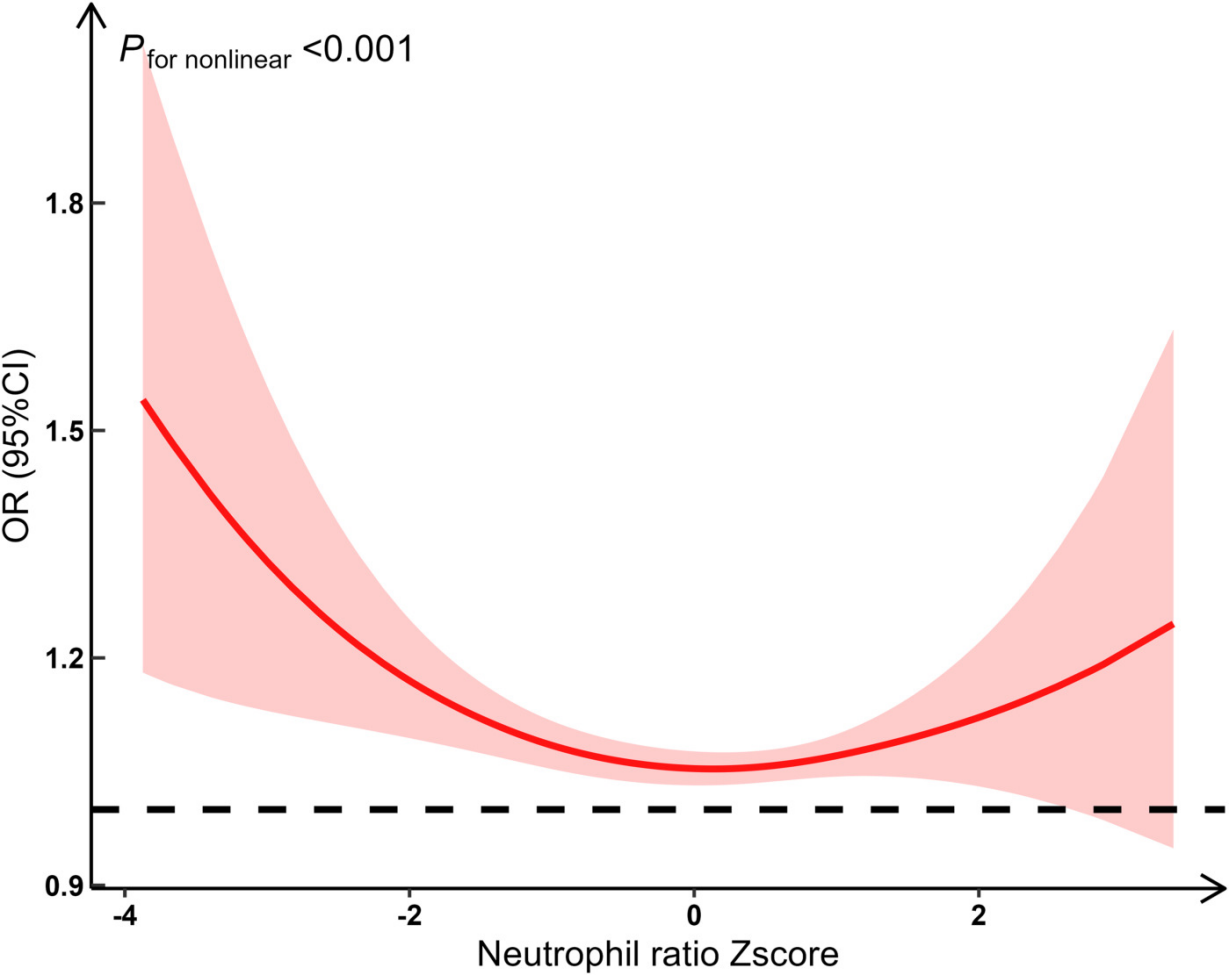
Restricted cubic spline regression result.

We fitted restricted cubic spline functions with 3–7 knots. Finally, 3 knots (located at the 10th, 50th, and 90th percentiles on the *x*-axis) were selected to capture potential nonlinear effects, with the lowest BIC (BIC was 329.07, 331.94, 335.74, 341.22 and 343.18, respectively). Using restricted cubic spline analysis with 3 knots, we found a significant nonlinear relationship between NR and the risk of poor outcomes (*F* = 13.27, *P* < 0.001). Specifically, the risk of poor outcomes significantly increased when the NR was either low or high, while the risk remained relatively stable at intermediate levels. This suggests that the relationship between NR and the risk of poor outcomes is not linear, potentially exhibiting a U-shaped relationship (Figure 3).

### Sensitivity analysis

Results were consistent with the primary results, when defining voluntary discharge as a poor outcome, and when using non-imputed data (Table 2, Supplementary Tables S4–S6).

## Discussion

In this study, we have made several important findings about NR and short-term prognosis of HIE. First, the NR over time could be classified into two classes: slowly declining stable group (Class 1) and increasing group (Class 2). Second, compared to the slowly declining stable group, the increasing group was a risk factor for short-term poor outcomes. The likelihood of short-term adverse outcomes in the increasing group was 4.78 times [OR_adj_ (95% CI): 4.78 (2.43, 9.27)] higher than that in the decreasing group. Third, there appeared to be a U-shaped relationship between NR and short-term poor outcomes.

Based on LGMM trajectory analysis, we found that NR exhibited dynamic changes over time and primarily manifested in two distinct patterns, a slowly declining stable group (Class 1) and a increasing (Class 2) group. And the increasing NR group was a risk factor for short-term poor outcomes in perinatal HIE, which was similar to findings by Povroznik et al. (7, 8). However, while their study provided a simple description of absolute number changes, we not only described dynamic changes but also classified them based on their different patterns of variation. Mechanisms underlying this association may include: (1) perinatal hypoxia–ischemia causeed a rapid increase of circulating neutrophils followed by pronounced infiltration into the injured brain parenchyma (11, 13, 23), and (2) Neonatal neutrophils were activated in the hypoxic-ischemic brain (11, 13). This discovery hold important implications for clinical practice, potentially providing a biomarker for assessing short-term prognosis in HIE patients. In clinical practice, an elevated NR as a prognostic indicator may aid in early identification of high-risk patients and guide personalized treatment decisions.

We observed a non-linear U-shaped relationship between NR and short-term poor outcomes of perinatal HIE. Elevated NR above standard levels increased the risk of poor HIE outcomes, consistent with previous findings (7, 8) that associate higher NR with exacerbated neurological damage progression. High NR may reflect an excessive inflammatory response, leading to poor prognosis. Toptan, showed neutrophil infiltration at early stages was crucial for exacerbating brain injury in newborns caused by hypoxic-ischemic, a condition in which inflammation heightens brain damage (24). Some studies observed an increased infiltration of neutrophils in the brain after exposure to HI (13, 15–19). Conversely, lower-than-standard NR also increased the risk of poor HIE outcomes, although there was limited literature directly linking low NR to HIE prognosis. Neutrophils played a positive role in defending against infections such as antibacterial (25), antifungal (26), and antiviral (27). And neutrophils had functions such as phagocytosing damaged tissues, clearing apoptotic cell debris, localizing the damage, and facilitating tissue regeneration and angiogenesis after injury (28). Reduced or insufficient neutrophils may limit these functions, thereby also leading to poor prognosis.

This study has several strengths. Firstly, to the best of our knowledge, this was the first study to use trajectory analysis to classify changes in the NR. Each category represented a different population, with significant association between the increasing NR group and short-term poor outcomes in perinatal HIE. Additionally, the study explored the non-linear U-shaped relationship between NR and HIE short-term outcomes. These founding may offer new insights into the diagnosis and treatment of HIE.

### Limitations

Our study also has several limitations. First, our study focused on analyzing the dynamic trajectory of NR during the first 3 postnatal weeks and its relationship with short-term HIE outcomes. It should be noted that our findings may not be directly applicable to clinical decision-making requiring immediate intervention within the critical first 72 h after birth. However, many studies have investigated the relationship between a single-time-point NR within 72 h after birth and HIE outcomes. These studies focused solely on the association between NR at a specific time point and HIE outcomes, whereas our research emphasizes the dynamic trend of NR changes in relation to short-term HIE outcomes, thereby forming a complementary perspective. Second, its retrospective design may introduce biases related to data collection and patient selection; however, our data were entirely based on EMRs, which are relatively objective and accurate. Third, as the study was conducted at a single institution, the findings may have limited generalizability; however, our institution treats the majority of HIE patients in the city and even the province. Four, maternal data were limited due to the nature of our institution as a children's hospital and the retrospective cohort design, making it challenging to trace and supplement this information. In the future, we plan to conduct a prospective study to collect and supplement detailed maternal medical history in real-time. Additionally, the focus on short-term prognosis means that longer-term outcomes were not evaluated. We have already established a follow-up system to monitor the long-term prognosis of such patients and plan to conduct further research.

## Conclusion

This study observed the significant role of the NR in short-term outcomes of perinatal HIE. Through trajectory analysis, we identified two distinct patterns of NR changes over time: a slowly declining stable group and an ascending group. Our findings revealed that the ascending NR group was strongly associated with poorer short-term outcomes. Additionally, we observed a U-shaped relationship between NR and short-term outcomes, indicating that both low and high NRs were associated with increased risk. These insights underscored the importance of monitoring NR dynamics in clinical practice to better assess prognosis and guide personalized treatment decisions for HIE patients. Future research should aim to validate these findings in larger, multi-center studies and explored the underlying mechanisms driving these associations.

## Data Availability

Anonymized individual participant data will be made available to researchers who provide a methodologically sound proposal.
