# Supplementary material for: Neutrophil ratio trajectory and the short-term outcome of perinatal hypoxic-ischemic encephalopathy
Source: Front Pediatr. 2025 Jun 17;13:1555981. doi: 10.3389/fped.2025.1555981 (PMC12209201; doi:10.3389/fped.2025.1555981)
Supplement: Supplementary file 1 [file Datasheet1.pdf]

Table S1 Results of univariate logistic regression regarding outcome groups

Variable	Main analysis		Sensitivity analysis（non-imputed data）	
	***OR*(95%*C*I)**	***P***	***OR*(95%*CI*)**	***P***
Ascending(Trajectory class 2)	5.72(2.97,10.87)	<0.001	3.79(1.90,7.32)	<0.001
Female	0.93(0.48,1.73)	0.82	0.93(0.48,1.73)	0.82
Birth Weight(kg)	0.43(0.29,0.64)	<0.001	0.42(0.28,0.64)	<0.001
GA(weeks)	0.87(0.80,0.96)	<0.001	0.87(0.79,0.95)	<0.001
Spesis(Yes)	1.28(0.69,2.34)	0.42	1.28(0.69,2.34)	0.42
Pregnancy Complications (Yes)	0.60(0.26,1.21)	0.18	0.63(0.28,1.28)	0.23
Medication during Pregnancy (Yes)	0.64(0.28,1.30)	0.24	0.67(0.30,1.37)	0.30
Parity				
1	1	-	1	-
2	0.75(0.32,1.73)	0.49	0.62(0.26,1.49)	0.29
≥3	1.05(0.51,2.24)	0.91	1.05(0.51,2.24)	0.91
Number of deliveries				
1	1	-	1	-
2	1.13(0.57,2.30)	0.73	1.03(0.52,2.12)	0.93
≥3	1.45(0.58,3.43)	0.41	1.45(0.58,3.43)	0.41
Prematurity (Yes)	1.69(0.86,3.18)	0.11	1.75(0.89,3.30)	0.09
Apgar score 1min	0.92(0.83,1.03)	0.14	0.96(0.85,1.07)	0.43
Apgar score 5min	0.99(0.87,1.14)	0.93	1.05(0.91,1.22)	0.54
Apgar score 10min	0.90(0.78,1.06)	0.18	0.89(0.75,1.07)	0.20
Delivery Type (C-section)	0.84(0.45,1.62)	0.60	0.82(0.44,1.57)	0.53
iUGR (Yes)	0.94(0.51,1.75)	0.84	0.99(0.53,1.90)	0.98

Premature Rupture of Fetal Membranes (Yes)	0.63(0.24,1.43)	0.31	0.66(0.25,1.49)	0.36
Amniotic fluid (bloody or polluted)	1.06(0.58,1.95)	0.85	1.15(0.61,2.20)	0.66
Placenta (Abnormal)	0.92(0.31,2.22)	0.87	0.96(0.32,2.32)	0.93
Father Age	1.00(0.95,1.06)	0.97	1.00(0.95,1.06)	0.92
Mother Age	1.03(0.98,1.09)	0.28	1.03(0.98,1.09)	0.27
Environment during Pregnancy(Village)	1.93(0.95,4.34)	0.09	1.82(0.89,4.10)	0.12
Hospital days	1.02(1.00,1.03)	0.07	1.02(1.00,1.03)	0.08
Hypothermia Treatment (Yes)	0.57(0.17,1.45)	0.29	0.57(0.17,1.45)	0.29

Table S2 Univariate logistic regression results with NR grouping as the outcome variable

**Variable**	**Main analysis**	
	***OR*(95%*CI*)**	***P***
Female	0.82(0.48,1.35)	0.45
Birth Weight(kg)	0.62(0.45,0.84)	<0.001
GA(weeks)	0.90(0.84,0.98)	0.01
Sepsis	1.46(0.91,2.35)	0.12
Pregnancy Complications (Yes)	1.47(0.88,2.41)	0.13
Medication during Pregnancy (Yes)	0.84(0.47,1.43)	0.54
Parity		
1		
2	1.90(0.99,3.81)	0.06
≥3	1.39(0.73,2.77)	0.33
Number of deliveries		
1		

2	1.32(0.78,2.30)	0.32
≥3	1.27(0.60,2.60)	0.52
Prematurity (Yes)	1.67(0.98,2.77)	0.05
Apgar score 1min	0.88(0.81,0.96)	0.003
Apgar score 5min	0.95(0.86,1.05)	0.29
Apgar score 10min	0.85(0.76,0.95)	0.01
Delivery Type (C-section)	1.40(0.83,2.46)	0.22
iUGR (Yes)	1.41(0.87,2.37)	0.17
Premature Rupture of Fetal Membranes (Yes)	0.67(0.33,1.27)	0.25
Amniotic fluid (bloody or polluted)	1.20(0.75,1.95)	0.45
Placenta (Abnormal)	1.12(0.52,2.21)	0.76
Father Age	1.04(0.99,1.08)	0.10
Mother Age	1.04(1.00,1.09)	0.06
Environment during Pregnancy(Village)	2.52(1.40,4.90)	0.004
Hospital days	1.03(1.01,1.05)	<0.001
Hypothermia Treatment (Yes)	0.66(0.28,1.35)	0.29

Table S3 Results of multivariate logistic regression regarding outcome groups

Variable	Main analysis			Sensitivity analysis（non-imputed data）		
	**n**	***OR*(95%*CI*)**	***P***	***n***	***OR*(95%*CI*)**	***P***
(Intercept)	594	0.23(0.00,11.35)	0.47	574	0.55(0.01,28.46)	0.77
Ascending(Trajectory class 2)	594	4.78(2.43,9.27)	<0.001	574	2.75(1.24,5.74)	<0.01
GA(weeks)	594	1.02(0.90,1.16)	0.76	574	1.01(0.88,1.16)	0.88
Birth Weight(kg)	594	0.47(0.26,0.82)	0.01	574	0.41(0.22,0.74)	<0.01
Sepsis	594	1.01(0..53,1.92)	0.97	574	0.93(0.47,1.82)	0.84
